# Enhancing wine fermentation through concurrent utilization of *Lachancea thermotolerans* and lactic acid bacteria (*Oenococcus oeni* and *Lactiplantibacillus plantarum*) or *Schizosaccharomyces pombe*

**DOI:** 10.1016/j.fochx.2024.102054

**Published:** 2024-11-30

**Authors:** Javier Vicente, Li Wang, Silvia Brezina, Stefanie Fritsch, Eva Navascués, Antonio Santos, Fernando Calderón, Wendu Tesfaye, Domingo Marquina, Doris Rauhut, Santiago Benito

**Affiliations:** a Unit of Microbiology, Genetics, Physiology and Microbiology Department, Biology Faculty, Complutense University of Madrid, Ciudad Universitaria, S/N, 28040 Madrid, Spain; b Department of Microbiology and Biochemistry, Hochschule Geisenheim University (HGU), Von-Lade-Straße 1, 65366 Geisenheim, Germany; c Department of Chemistry and Food Technology, Polytechnic University of Madrid, Ciudad Universitaria, S/N, 28040 Madrid, Spain

**Keywords:** *Lachancea thermotolerans*, *Oenococus oeni*, *Lactiplantibacillus plantarum*, *Lactobacillus plantarum*, *Saccharomyces*, *Schizosaccharomyces pombe*, Malic acid, Lactic acid, L-Lactic acid (PubChem CID107689), L-Malic acid (PubChem CID222656), Acetic acid (PubChem CID176), Succinic acid (PubChem CID1110), Ethyl lactate (PubChem CID7344), Phenylethyl Alcohol (PubChem CID6054), Diacetyl (PubChem CID6061)

## Abstract

Most commercially available red wines undergo alcoholic fermentation by *Saccharomyces* yeasts, followed by a second fermentation with the lactic acid bacteria *Oenococcus oeni* once the initial process is complete. However, this traditional approach can encounter complications in specific scenarios. These situations pose risks such as stalled alcoholic fermentation or the growth of undesirable bacteria while the process remains incomplete, leaving residual sugars in the wine. To address these challenges and the issue of low acidity prevalent in warmer viticultural regions, several novel alternatives are available. The alternatives involve the combined use of *Lachancea thermotolerans* to increase the acidity of the musts, lactic acid bacteria (*Oenococcus oeni* and *Lactiplantibacillus plantarum*) to ensure malic acid stability during early alcoholic fermentation stages, and *Saccharomyces cerevisiae* to properly complete alcoholic fermentation. The study showed variations in the final chemical parameters of wines based on the microorganisms used.

## Highlights

• Fermentations involving L. thermotoleans, Saccharomyces, Lactic bacteria and S.pombe.
• Some biotechnologies involving the selected microorganisms are compared for the first time.
• Specific chemical compounds are reported for the first time comparing those biotechnologies.
• The proposed methodology comparing different biotechnologies is reproducible.
• The proposed biotechnologies improve several wine quality chemical paramteres.

## 1 Introduction

Numerous viticultural regions face challenges concerning grape musts that demonstrate potential impediments to traditional sequential malolactic fermentation after alcoholic fermentation. Some of these issues encompass elevated sugar concentrations, inadequate nutrient levels, or reduced acid content, culminating in a pH nearing 4. Under such circumstances, alcoholic fermentation may extend beyond several weeks or even months, occasionally experiencing sluggishness or cessation. These conditions foster a milieu conducive to the proliferation of undesirable spontaneous spoilage microorganisms, including wild lactic acid bacteria (Sumby et al., 2014) or *Brettanomyces* spp. (Pinto et al., 2020), detrimentally impacting wine quality by elevating acetic acid, volatile phenols, or other undesirable compounds. Previous studies have proposed potential microbiological remedies, such as controlled simultaneous alcoholic and malolactic fermentation (Knoll et al., 2012; Urbina et al., 2021), or the utilization of alternative microorganisms proficient in metabolizing malic acid (Benito, 2019).

*Lachancea thermotolerans,* a popular non-*Saccharomyces* yeast in warm viticultural regions (Jolly et al., 2014), enhances must quality elevating acidity through lactic acid production during alcoholic fermentation (Benito, 2018; Capozzi et al., 2021; Fairbairn et al., 2021; Hranilovic et al., 2021, Hranilovic et al., 2022; Porter, Divol and Setati, 2019a, Porter, Divol and Setati, 2019b; Vilela, 2019). This acid is chemically stable, unlike tartaric acid, and is generated by *L. thermotolerans* from sugar metabolism during alcoholic fermentation (Vicente et al., 2021). Unlike lactic acid bacteria, its final concentration does not rely on the initial malic acid concentration of the grape juice, since it is produced from the sugars present in grape must (Benito, 2018). Previous studies have reported lactic acid increases up to 10 g/L and pH reductions to 0.55 in sequential fermentations involving L. *thermotolerans* with *Saccharomyces cerevisiae* (Hranilovic et al., 2021). Additionally, modern literature highlights other secondary virtues of *L. thermotolerans*, including aroma enhancements (Borren & Tian, 2020; Escribano et al., 2018), minimal acetic acid production (Vilela, 2018), ethanol reduction (Hranilovic et al., 2018), increased glycerol (Comitini et al., 2011; Gobbi et al., 2013; Kapsopoulou et al., 2007), lowered acetaldehyde (Ciani et al., 2006), improved color (Hranilovic et al., 2018), and polysaccharide increase (Comitini et al., 2011; Domizio et al., 2014; Gobbi et al., 2013).

The concurrent action of yeast and lactic acid bacteria during alcoholic fermentation presents an alternative to traditional malolactic fermentation (Bartowsky et al., 2015; Du Toit et al., 2011; Mendes Ferreira & Mendes-Faia, 2020; Pardo & Ferrer, 2018; Petruzzi et al., 2017), particularly in demanding conditions such as nutrient-deficient, high-sugar or high-pH grape juices (Krieger-Weber et al., 2020; Virdis et al., 2021). Among the lactic acid bacteria studied in winemaking, *Oenococcus oeni* and *Lactiplantibacillus plantarum* (former *Lactobacillus plantarum*) have garnered the most attention. In challenging circumstances, like prolonged alcoholic fermentation, *O. oeni* might consume residual sugars, leading to increased acetic acid and diacetyl concentrations (Sumby et al., 2014). Moreover, issues such as the generation of biogenic amines are heightened, especially in uncontrolled spontaneous malolactic fermentations conducted with unselected wild strains (Benito, 2019; Blanco et al., 2020). Conversely, selectively chosen strains of *L. plantarum* demonstrate a facultative heterofermentative nature, selectively metabolizing malic acid in musts without impacting sugars or increasing volatile acidity (Krieger-Weber et al., 2020). This characteristic allows for early stabilization of malic acid during alcoholic fermentation, aiding in safeguarding against bacteria or spoilage microorganisms upon achieving stability. As a result, there has been growing interest in simultaneous alcoholic and malolactic fermentation to streamline production timelines and reduce the risk of deviations. However, a significant limitation of *L. plantarum* is its moderate sensitivity to ethanol, which makes it most suitable for use during the early stages of alcoholic fermentation, before ethanol concentrations become high, especially in grape musts with elevated pH levels (Pardo & Ferrer, 2018).

This study proposes an alternative method to prevent and manage possible challenging alcoholic fermentations of grape musts by employing combined malolactic and alcoholic fermentations using selected strains of yeast and bacteria. This approach aims to enhance acidity levels, potentially improving sensory characteristics by increasing acidity without encountering the negative consequences associated with problematic fermentation endings, such as heightened volatile acidity. More specifically, the use of *L. thermotolerans* is shown to raise lactic acid levels and lower pH values during alcoholic fermentation. Meanwhile, bacteria as *O. oeni* or *L. plantarum* are utilized to consume malic acid, ensuring the microbial stability of wine in the early stages of alcoholic fermentation, and preventing challenging malolactic fermentations, especially in environments with high ethanol and residual sugar content. This approach aids in managing undesirable bacterial growth in the later stages of alcoholic fermentation. Additionally, *S. cerevisiae* is employed to ensure proper completion of alcoholic fermentation. Moreover, the study includes control experiments involving a contemporary biotechnological approach with similar objectives, centered on the combination of *L. thermotolerans* and *Schizosaccharomyces pombe*.

**Hypothesis:** The hypothesis of this study proposes that the use of *L. thermotolerans* in combination with other microorganisms, such as *O. oeni*, *S. pombe*, and *S. cerevisiae*, can optimize fermentation outcomes, particularly under challenging conditions like those encountered in warm viticultural regions affected by climate change. To explore this, the study focuses on analyzing a broad set of chemical and volatile compounds. Specifically, it examines four key organic acids (L-malic acid, L-lactic acid, succinic acid, and acetic acid), which are crucial for understanding the metabolic activity of the microorganisms and their influence on acidity, stability, and balance of the wine. Additionally, two alcohols (ethanol and glycerol) are analyzed, as they can impact the body of wine, texture, and alcohol content. The study also evaluates ten chemical parameters, including pH, total acidity, color intensity, polyphenol index, and the CIELAB color coordinates, which provide insights into the visual characteristics of wine, acidity, and overall stability. Moreover, the analysis includes two sugars (glucose and fructose) to evaluate fermentation progress and ensure efficient sugar consumption. Lastly, sixteen volatile compounds, responsible for the aroma profile of wine, are studied to assess the complexity of the wine. By investigating these compounds and parameters across different microbial fermentation scenarios, the hypothesis suggests that these combinations can enhance fermentation efficiency, wine stability, and sensory quality, offering solutions to common challenges such as low acidity, high ethanol levels, and undesirable fermentation outcomes, thereby improving overall wine quality in warmer climates.

## 2 Material and methods

### 2.1 Microorganisms and vinification assays

This study used the following microorganisms: *Lachancea thermotolerans* MJ-311 (Complutense University of Madrid, Madrid, Spain), *Oenococcus oeni* Lalvin VP41 (Lallemand, Montreal, Canada), *Lactiplantibacillus plantarum* ML Prime (Lallemand, Montreal, Canada), *Saccharomyces cerevisiae* AG006 (Agrovín S.L, Alcazar de San Juan, Spain) and *Schizosaccharomyces pombe* Atecrem 12H (Bioenologia, Oderzo, Italy).

Yeast strains were precultured in 100 mL of YMB (0.5 % peptone, 0.3 % malt extract, 0.3 % yeast extract and 1.0 % glucose) in 250 mL bottles and incubated at 28 °C with shaking at 150 rpm for 24 h, and its cellular concentration was determined by measuring the optical density (O.D., 600 nm) (Genesys 2.0 Spectrophotometer, ThermoFisher, Waltham, MA, USA). Fermentation cultures were inoculated at a concentration of 10^6^ cells/mL (≈ 0.2 OD) for each yeast strain. For lactic acid bacteria, commercially available products were used in accordance with the inoculum recommended by the manufacturer to achieve a final concentration of 10^6^ cells/mL under aseptic conditions, within a Telstar Mini-H laminar flow hood (Telstar S.A., Madrid, Spain), using sterilized distilled water.

Vinifications were conducted using a commercial *Vitis vinifera* L. Tempranillo grape juice, marketed as CarrefourBio (Carrefour España, Madrid, Spain), with a pH of 3.5, malic acid content of 1.48 g/L, and lactic and acetic acid levels below 0.1 g/L. The grape juice was supplemented with 0.30 g/L of Actimax Natura (Agrovin S.A., Alcázar de San Juan, Spain) and enriched to 215 g/L with an equimolar mixture of glucose and fructose (Fisher Scientific, Pittsburgh, USA). All preparations were carried out under strict aseptic conditions within a Telstar Mini-H laminar flow hood (Telstar S.A., Madrid, Spain). The final nitrogen concentrations were 198 mg/L for primary amino nitrogen and 32 mg/L for ammonia nitrogen.

The fermentations were carried out in sterilized 250 mL Pyrex™ borosilicate glass reagent bottles (Fisherbrand, Pittsburgh, USA) filled up to 210 mL under rigorous aseptic conditions within a Telstar Mini-H laminar flow hood (Telstar S.A., Madrid, Spain). Each fermentation vessel was equipped with a partially open polypropylene cap and pouring ring, enabling CO_2_ release while averting microbial contamination. These fermentations were replicated three times at 25 °C using a Zanotti Ecology climate-controlled chamber (Zanotti, Pieve di Soligo, Italy).

Fermentative kinetics of alcoholic fermentations were monitored by measuring weight loss every 24 h, and fermentations were considered complete when the weight loss was less than 0.01 % per day. After fermentation, all wines were centrifuged (7000 rpm for 5 min) and stored at 4 °C until further analysis.

Ten distinct treatments were administered, with Table 1 outlining the combinations of species employed in each treatment and the sequential inoculation timings.

**Table 1:** Species combinations used in each treatment. SC: *S. cerevisiae*, OE: *O. oeni*, LP: *L. plantarum*, LT: *L. thermotolerans*, SP: *S. pombe*. *: 24 h, “…”: 72 h, “….”: end or alcoholic fermentation.

SC	Inoculation of the must with *S. cerevisiae* (10^6^ CFU/mL) alone.
SC….OE	Inoculation of the must with *S. cerevisiae* (10^6^ CFU/mL) followed by *O. oeni* (10^6^ CFU/mL) after alcoholic fermentation.
LT…SC	Inoculation of the must with *L. thermotolerans* (10^6^ CFU/mL) followed by *S. cerevisiae* (10^6^ CFU/mL) 72 h later.
LT…SC….OE	Inoculation of the must with *L. thermotolerans* (10^6^ CFU/mL) followed by *S. cerevisiae* (10^6^ CFU/mL) 72 h later, followed by *O. oeni* (10^6^ CFU/mL) after alcoholic fermentation.
LT*OE…SC	Inoculation of the must with *L. thermotolerans* (10^6^ CFU/mL) followed by *O. oeni* (10^6^ CFU/mL) 24 h later and followed by *S. cerevisiae* (10^6^ CFU/mL) 72 h later.
LT*LP…SC	Inoculation of the must with *L. thermotolerans* (10^6^ CFU/mL) followed by *L. plantarum* (10^6^ CFU/mL) 24 h later and followed by *S. cerevisiae* (10^6^ CFU/mL) 72 h later.
SC*LP	Inoculation of the must with *S. cerevisiae* (10^6^ CFU/mL) followed by *L. plantarum* (10 CFU/mL) 24 h later.
SC*OE	Inoculation of the must with *S. cerevisiae* (10^6^ CFU/mL) followed by *O. oeni* (10 CFU/mL) 24 h later.
SP	Inoculation of the must with *S. pombe* (10^6^ CFU/mL) alone.
LT…SP	Inoculation of the must with *L. thermotolerans* (10^6^ CFU/mL) followed by *S. pombe* (10^6^ CFU/mL) 72 h later.

In sequential yeast fermentations, the more fermentative yeast species (*S. cerevisiae* or *S. pombe*) was inoculated 72 h (3 days) after the initial inoculation of *L. thermotolerans*. In mixed fermentations involving yeast and lactic acid bacteria, lactic acid bacteria were introduced 24 h after the initial yeast inoculation. Sequential malolactic fermentations (SC….OE; LT..SC…OE) followed alcoholic fermentation in sterilized 100 mL Pyrex™ borosilicate glass reagent bottles (Fisherbrand, Pittsburgh, USA) totally filled without air space under rigorous aseptic conditions within a Telstar Mini-H laminar flow hood (Telstar S.A., Madrid, Spain). The sequential malolactic fermentations (SC…OE; LT…SC…OE) were carried out at 18 °C using an FTC 90E Refrigerated Incubator (Velp Scientifica, Usmate Velate, Italy) until the malic acid was completely consumed. The concentration of malic acid was monitored every 48 h using enzymatic analysis with a Miura Micro analyzer and an enzymatic kit (TDI, Barcelona, Spain). To track the evolution of malic acid, 0.5 mL samples were collected under strict aseptic conditions within a Telstar Mini-H laminar flow hood (Telstar S.A., Madrid, Spain).

### 2.2 Basic oenological parameters determinations

An enzymatic autoanalyzer Miura Micro and its enzymatic kits (TDI, Barcelona, Spain) were used to perform determinations of glucose and fructose, L-malic acid, L-lactic acid, acetic acid, primary amino nitrogen, and ammonia nitrogen. The determination of ethanol, total acidity, succinic acid, pH, glycerol, color intensity and CIELAB coordinates in the resulting wines were conducted using an autoanalyzer FTIR Bacchus 3 MultiSpec (TDI, Barcelona, Spain). pH was measured with a Crison pH Meter Basic 20 (Crison, Barcelona, Spain). Total tannin and total anthocyanin concentrations were determined according to the methods of Ribereau-Gayon et al. (2006) (Chen et al., 2018).

### 2.3 Volatile compounds determination

The analysis of esters, higher alcohols, and fatty acids was performed using the method developed by the Department of Microbiology and Biochemistry at HGU, as previously described (Jung et al., 2021).

For the analysis of diacetyl (2,3-butanedione), headspace injection combined with gas chromatography and mass spectrometry (HS-GC–MS) was employed, following the specified protocol. 5 mL of each sample was pipetted into a 10 mL amber headspace vial containing 1.7 g NaCl. 2,3-hexanedione (final concentration in the sample: 8 mg/L) and methanol‑*d*_3_ (final concentration in the sample: 20 mg/L) were added as internal standards. Finally, the vial was sealed airtight with a magnetic screw cap. For analysis a gas chromatograph GC 7890 combined with a mass spectrometer MS 5977B (Agilent Technologies, Santa Clara, USA) was used, which was equipped with an automated headspace sampling system (Multipurpose Sampler Robotic, Gerstel, Mülheim an der Ruhr, Germany). The sample was incubated for 30 min at 80 °C. Subsequently 400 μL of sample gas volume (syringe temperature: 65 °C) were injected into a cooled injection system at 10 °C, ramped to 220 °C with 12 °C/s (CIS-4, Gerstel, Mülheim an der Ruhr, Germany) in split mode (1:5). For chromatographic separation a Stabilwax-DA capillary column (30 m × 0.25 mm I. D., 0,25 μm film thickness; Restek, Bad Homburg, Germany) was used with helium as carrier gas in constant flow (1,2 mL/min) and the following oven temperature program: 30 °C (10 min), 10 °C/min to 240 °C (5 min). The mass spectrometer (MS transfer line 250 °C, MS source 230 °C, MS quadrupol 150 °C) was set to SIM mode for detection: methanol‑*d*_3_: *m*/*z*: 30, 33, 35; diacetyl: m/z: 42, 43, 86; 2,3-hexanedione: m/z: 43, 71, 114. Calibration was done by means of standard addition in white wine (Riesling clone GM 64, vintage 2021, Department of Grapevine Breeding, HGU).

### 2.4 Statistical analyses

All statistical analyses were conducted using R software version 4.1.2 (R Development Core Team, 2013). Analysis of variance (ANOVA) and Tukey post-hoc tests were utilized to compare the different groups and values.

## 3 Results and discussion

### 3.1 Glucose and fructose

**Fig. 1:**
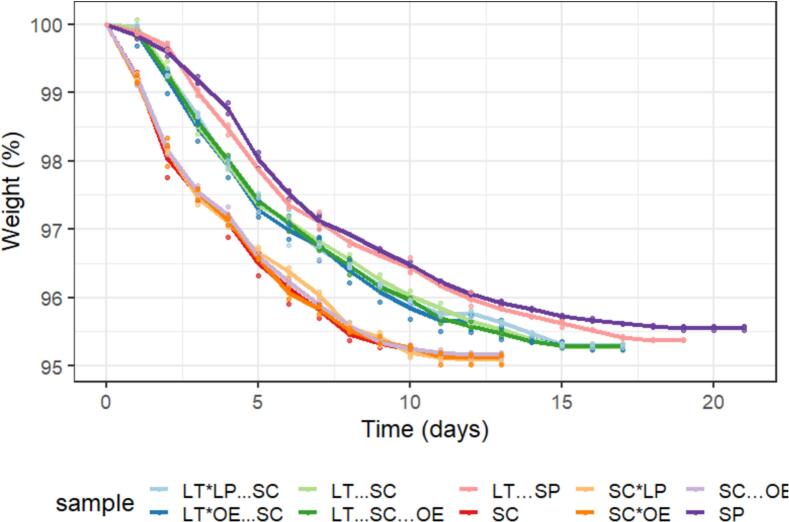
Fermentation kinetics of gravimetrically measured variants by total weight loss during alcoholic fermentation of: *S. cerevisiae* alone (SC); sequential fermentation with *S. cerevisiae* and *O. oeni* (SC…OE); sequential fermentation with *L. thermotolerans* and *S. cerevisiae* (LT…SC); sequential fermentation with *L. thermotolerans* and *S. cerevisiae*, followed by *O. oeni* (LT…SC…OE); sequential fermentation with *L. thermotolerans* and *S. pombe* (LT…SP); *S. pombe* alone (SP); sequential fermentation with *L. thermotolerans* and *O. oeni*, followed by *S. cerevisiae* (LT*OE…SC); sequential fermentation with *L. thermotolerans* and *L. plantarum*, followed by *S. cerevisiae* (LT*LP…SC); fermentation with *S. cerevisiae* followed by *O. oeni* 1 day after (SC*OE); fermentation with *S. cerevisiae* followed by *L. plantarum* 1 day after (SC*LP).

All concluding fermentations achieved final glucose and fructose concentrations below 2 g/L (Table 2), indicating the successful completion of all alcoholic fermentations. Nevertheless, fermentation kinetics showed different fermentation times depending on the inoculation strategy (Fig. 1). Alcoholic fermentations involving *S. cerevisiae* fermented faster finishing in 12 days while sequential fermentations involving non-*Saccharomyces* required between 4 and 8 additional days to finish the alcoholic fermentation. Previous studies have shown that *L. thermotolerans* typically cannot ferment beyond 10 % (*v*/v) ethanol concentrations (Benito, 2018; Vicente et al., 2021). Nevertheless, when paired with more fermentative yeast species, such as those from the genera *Saccharomyces* or *Schizosaccharomyces*, alcoholic fermentations typically reach completion effectively, especially in the production of standard industrial dry wines (Benito, 2020). The findings of this study align with and support these previous observations.

**Table 2:** Final basic chemical analysis of fermentations: *S. cerevisiae* alone (SC); sequential fermentation with *S. cerevisiae* and *O. oeni* (SC….OE); sequential fermentation with *L. thermotolerans* and *S. cerevisiae* (LT…SC); sequential fermentation with *L. thermotolerans* and *S. cerevisiae*, followed by *O. oeni* (LT…SC….OE); sequential fermentation with *L. thermotolerans* and *S. pombe* (LT…SP); *S. pombe* alone (SP); sequential fermentation with *L. thermotolerans* and *O. oeni*, followed by *S. cerevisiae* (LT*OE…SC); sequential fermentation with *L. thermotolerans* and *L. plantarum*, followed by *S. cerevisiae* (LT*LP…SC); fermentation with *S. cerevisiae* followed by *O. oeni* 1 day after (SC*OE); fermentation with *S. cerevisiae* followed by *L. plantarum* 1 day after (SC*LP).

Species combinations	Ethanol (%)	pH	Total Acidity (g/L)	Acetic acid (g/L)	Malic acid (g/L)	Lactic acid (g/L)	Succinic acid (g/L)	Glucose + Fructose (g/L)	Glycerol (g/L)
SC	12.53 ± 0.02d	3.53 ± 0.01 cd	5.57 ± 0.05d	0.18 ± 0.02d	1.22 ± 0.05e	0.00 ± 0.00a	0.57 ± 0.02b	1.34 ± 0.20ef	4.58 ± 0.01bcd
SC….OE	12.49 ± 0.07d	3.62 ± 0.01e	4.76 ± 0.03b	0.34 ± 0.02e	0.00 ± 0.00a	0.68 ± 0.03c	0.43 ± 0.04a	0.48 ± 0.08abc	4.61 ± 0.08bcd
LT…SC	12.35 ± 0.05d	3.45 ± 0.01a	6.34 ± 0.19e	0.09 ± 0.03ab	0.83 ± 0.09d	1.46 ± 0.18d	0.78 ± 0.05de	0.65 ± 0.18bc	4.62 ± 0.24bcd
LT…SC….OE	12.11 ± 0.05bc	3.52 ± 0.04bcd	5.78 ± 0.11d	0.15 ± 0.01 cd	0.00 ± 0.00a	1.90 ± 0.12e	0.60 ± 0.05bc	1.29 ± 0.11e	4.83 ± 0.30d
LT*OE…SC	12.10 ± 0.05bc	3.48 ± 0.02ab	6.17 ± 0.09e	0.31 ± 0.03e	0.00 ± 0.00a	2.37 ± 0.04f	0.71 ± 0.06cde	0.37 ± 0.21ab	4.04 ± 0.13a
LT*LP…SC	12.05 ± 0.06b	3.50 ± 0.02bc	6.11 ± 0.12e	0.12 ± 0.03bc	0.56 ± 0.10c	1.55 ± 0.04d	0.83 ± 0.05e	0.20 ± 0.15a	4.31 ± 0.21ab
SC*LP	11.97 ± 0.07ab	3.56 ± 0.02d	5.25 ± 0.08c	0.16 ± 0.03 cd	0.63 ± 0.04c	0.41 ± 0.03b	0.54 ± 0.09ab	1.12 ± 0.22de	4.48 ± 0.05bcd
SC*OE	12.06 ± 0.08b	3.64 ± 0.01e	4.77 ± 0.04b	0.30 ± 0.02e	0.06 ± 0.06ab	0.60 ± 0.06bc	0.41 ± 0.04a	0.82 ± 0.28 cd	4.40 ± 0.12bc
SP	12.32 ± 0.13 cd	3.71 ± 0.02f	4.32 ± 0.18a	0.09 ± 0.03ab	0.11 ± 0.04b	0.00 ± 0.00a	1.07 ± 0.15f	0.31 ± 0.13ab	5.40 ± 0.39e
LT…SP	11.78 ± 0.35a	3.54 ± 0.03 cd	5.58 ± 0.28d	0.06 ± 0.04a	0.05 ± 0.03ab	1.60 ± 0.31d	0.70 ± 0.08 cd	1.68 ± 0.36f	4.74 ± 0.25 cd

Results are mean ± SD of three replicates. Different letters indicate statistical significance between groups.

### 3.2 Ethanol

Fermentations exclusively involving a blend of *L. thermotolerans* and *S. pombe* exhibited statistically significant distinctions compared to the *S. cerevisiae* control. The synergy between these two non-*Saccharomyces* strains resulted in a final wine with a reduced ethanol concentration by 0.75 % (*v*/v) in comparison to the *S. cerevisiae* control. Prior investigations have noted this occurrence and attributed it to the lactic acid metabolism of *L. thermotolerans*, which diverts carbon atoms away from the ethanol metabolism typical in alcoholic fermentation (Vicente et al., 2021).

Previous studies have reported a decrease in final ethanol concentration of 0.62 % (v/v) when comparing the traditional winemaking method—in which alcoholic fermentation is completed first, followed by malolactic fermentation (SC…OE)—with co-inoculation of *L. thermotolerans*, *O. oeni*, and *Saccharomyces* (LT*OE…SC) (Urbina et al., 2021)*.* Another study observed ethanol reductions ranging from 1.12 % to 0.09 % (v/v) (Snyder et al., 2021), depending on the specific strain of *L. thermotolerans* used. Strains that produced the highest lactic acid concentrations showed the most significant ethanol reductions. In the present study, the ethanol reduction was 0.39 % (v/v) (Table 2)*.* This difference increased from 0.39 % to 0.44 % (v/v) when *L. plantarum (*LT*LP…SC) was used instead of *O. oeni* (LT*OE…SC). The ethanol reduction achieved with the combined use of L. *thermotolerans* and *S. pombe* (LT…SP) compared to the traditional winemaking method (SC…OE) was 0.71 % (v/v). Previous studies using this biotechnology have reported ethanol reductions ranging from 0.4 % to 1.27 % (Benito, 2020).

### 3.3 Malic acid

The consumption of malic acid varied across all fermentations starting with an initial value of 1.48 g/L in the grape juice (Table 2). When combined with *O. oeni*, mixed fermentation resulted in a complete 100 % consumption of the initial malic acid, consistent with previous findings (Du Plessis et al., 2019; Snyder et al., 2021; Urbina et al., 2021). However, different studies indicate that the reduction level varies based on the specific strains of *L. thermotolerans* and *O. oeni* used (Snyder et al., 2021). Nevertheless, it should be noted that when *L. thermotolerans* rapidly produces high concentrations of lactic acid at the onset of alcoholic fermentation, it can inhibit the performance of *O. oeni* or *L. plantarum* (Vicente et al., 2022).

Fermentations that involved a combination of *L. thermotolerans* and *S. pombe* showed a consumption rate of 97 % of the initial malic acid. That reduction rate is close to those of mixed fermentations using yeasts and *O. oeni*. Most studies have reported reductions close to 100 % when combining *L. thermotolerans* and *S. pombe* (Benito, 2020), while others show more moderate consumptions at around 50 % (Wang et al., 2019). These strategies are especially relevant in situations where simultaneous alcoholic and malolactic fermentations could lead to increased volatile acidity (Benito, 2020; Vicente et al., 2022). This is particularly the case when both fermentations occur concurrently in a sluggish state, potentially raising volatile acidity due to the metabolic activity of lactic acid bacteria on residual sugars.

Pure *S. pombe* fermentation resulted in the consumption of 95 % of the initial concentration of malic acid. Previous studies have reported *S. pombe* consuming malic acid at rates ranging from 50 % to 100 % (Benito, 2019). Interestingly, one study observed higher malic acid degradation when combining *S. pombe* with specific *L. thermotolerans* strains compared to pure *S. pombe* fermentation (Vicente et al., 2023). In that study, the chosen *L. thermotolerans* strain was selected not only to produce lactic acid but also to consume over 50 % of the initial malic acid. In this study, Mixed fermentation involving *L. thermotolerans* and *S. cerevisiae* consumed approximately 44 % of the initial malic acid, while pure *S. cerevisiae* consumed 18 %. Most *L. thermotolerans* strains exhibit about 20 % malic acid consumption (Benito, 2018), although certain specific strains may reach 50–60 % (Blanco et al., 2020; Vicente et al., 2021), which is valuable in red wine production. Previous studies have also indicated that specific strains of *S. cerevisiae* can reduce up to 50 % of malic acid (Vicente et al., 2022). However, most *S. cerevisiae* strains tend to consume lower concentrations of malic acid, typically ranging from 10 % to 20 %. (Vicente et al., 2022).

Fermentations that included the lactic acid bacteria *L. plantarum* resulted in a notable decrease in malic acid levels by 58–62 % (Table 2). Previous studies also report similar reductions of approximately 50 % (Gardoni et al., 2021), while other research indicates higher reductions of up to 100 % (Vicente et al., 2022). Additionally, more significant malic acid breakdown, reaching up to 100 %, has been observed when combining *L. thermotolerans* and *L. plantarum* (Urbina et al., 2021). Notably, when the original grape juice had a pH of 4, *L. plantarum* exhibited enhanced performance (Urbina et al., 2021).

### 3.4 Lactic acid

In all fermentations involving *L. thermotolerans* or lactic acid bacteria (*O. oeni* or *L. plantarum*), lactic acid was significantly increased (Table 2). Conversely, pure fermentations carried out with *S. cerevisiae* or *S. pombe* did not yield any lactic acid due to the absence of the capability in these microorganisms to produce this acid. Notably, fermentations involving *L. thermotolerans* exhibited the highest final concentrations of lactic acids, ranging from 1.46 g/L to 2.37 g/L (Table 2). Combined sequential fermentation of *L. thermotolerans* and *O. oeni* (LT*OE…SC) resulted in a significantly higher production of lactic acid compared to the simple combination of *L. thermotolerans* and *S. cerevisiae* (LT…SC). This observation aligns with prior studies that have discussed similar phenomena based on the malic acid metabolism and sugar metabolism of *O. oeni* (Urbina et al., 2021). Previous works have documented a spectrum of lactic acid production by *L. thermotolerans*, varying from 0 to 6 g/L (Benito, 2018; Vicente et al., 2024). This variability is influenced by several factors including the specific strain of *L. thermotolerans*, its combination with *S. cerevisiae* strains, the duration of pure fermentation, and other pertinent factors (Vicente et al., 2021; Vicente et al., 2022). Studies have also noted lactic acid production in sequential combined fermentations involving L. *thermotolerans*, *O. oeni*, and *S. cerevisiae*, ranging from 0.7 g/L (Du Plessis et al., 2019) to 2.91 g/L (Urbina et al., 2021) when all malic acid was consumed. Furthermore, in comparisons made between combined fermentations involving *L. thermotolerans* and *S. pombe* versus those between *L. thermotolerans* and *S. cerevisiae*, similar levels of lactic acid production were observed (Table 2).

### 3.5 Succinic acid

Fermentations involving *L. thermotolerans* or *S. pombe* demonstrated higher final concentrations compared to their *S. cerevisiae* controls. Furthermore, sequential malolactic fermentations conducted by *O. oeni* resulted in a reduction of final concentrations from 0.12 to 0.14 g/L, whereas simultaneous malolactic fermentations decreased it from 0.07 to 0.16 g/L. Pure fermentation of *S. pombe* yielded the highest final concentration of 1.07 g/L, followed by combined fermentation of *L. thermotolerans* at 0.78 g/L, while pure *S. cerevisiae* fermentation produced 0.57 g/L (Table 2). Previous studies have highlighted both *S. pombe* and *L. thermotolerans* as superior producers of succinic acid compared to *S. cerevisiae* (Vicente et al., 2021; Vicente et al., 2022). This attribute holds interest in enhancing the minerality profile of specific wine types (Vicente et al., 2021), as it serves as a pivotal descriptor for certain wine appellations.

### 3.6 Acetic acid

The use of *O. oeni* in fermentations resulted in significantly higher final concentrations of acetic acid compared to the respective controls that did not undergo malolactic fermentation. In fermentations conducted solely with *S. cerevisiae*, the increase in acetic acid after sequential malolactic fermentation was 0.16 g/L, while for simultaneous malolactic fermentations, the increase was 0.12 g/L. On the contrary, in mixed fermentations involving *L. thermotolerans* and *S. cerevisiae*, the acetic acid increases were 0.06 g/L for sequential malolactic fermentation and 0.22 g/L for simultaneous malolactic fermentation. However, all final acetic acid concentrations remained below the recognized fault threshold of approximately 0.8 g/L. This observed increase aligns with previous research findings (Du Plessis et al., 2019; Urbina et al., 2021), although contrary observations exist (Snyder et al., 2021). This phenomenon is commonly attributed to the heterofermentative activity of *O. oeni* on fermentable sugars. *L. thermotolerans*-involved fermentations produced approximately 50 % less final acetic acid compared to standard *S. cerevisiae* controls. Previous studies have shown that *L. thermotolerans* generates less acetic acid than *S. cerevisiae*, although strain-specific variations exist (Benito, 2018; Vicente et al., 2021; Vilela, 2018). Fermentations with *S. pombe* exhibited the lowest concentrations of acetic acid, despite the species being known for high acetic acid production. However, recent studies show that select strains can produce moderate to low concentrations, and the specific strain used in this study was chosen for this characteristic (Benito, 2019).

### 3.7 pH

The sole fermentation of *S. pombe* exhibited the highest final pH of 3.71, whereas sequential fermentation involving *L. thermotolerans* and *S. cerevisiae* demonstrated the lowest final pH of 3.45. Sequential malolactic fermentation induced pH increases ranging from 0.07 to 0.09 units compared to their respective original controls.

Prior research has highlighted a pH reduction of 0.23 in winemaking trials that used a co-inoculation strategy with *L. thermotolerans*, *O. oeni*, and *Saccharomyces* (LT*OE…SC), as compared to the traditional method where alcoholic fermentation precedes malolactic fermentation (SC…OE) (Urbina et al., 2021). Variation in pH reduction has also been noted in another study for the same comparison, depending on the specific L. *thermotolerans* strains used, with reported changes ranging from 0 to 0.61 (Snyder et al., 2021). Strains producing the highest lactic acid concentrations resulted in the greatest pH decreases. In this study, the co-inoculation approach achieved a pH reduction of 0.14 (*v*/v) (Table 2). Using *L. plantarum* instead of *O. oeni* (LT*LP…SC) slightly reduced the pH difference from 0.14 to 0.12, though this change was not statistically significant. When applying a different combination of *L. thermotolerans* with *S. pombe* (LT…SP), a pH decrease of 0.08 was observed compared to the traditional method. Previous studies exploring similar biotechnological approaches have found pH reductions ranging from 0.07 to 0.5 (Benito, 2020).

### 3.8 Glycerol

The fermentation of pure *S. pombe* demonstrated the highest recorded glycerol concentration, reaching up to 5.4 g/L. Sequential combinations involving *L. thermotolerans* with either *S. cerevisiae* or *S. pombe* resulted in glycerol concentrations ranging from 4.61 to 4.83 g/L (Table 2). Previous research has identified the *Schizosaccharomyces* genus as a superior producer of glycerol compared to *Saccharomyces*, although strain variability within both genera has been observed, similar to the diversity found within *S. cerevisiae* strains (Benito, 2019; Benito, 2020). A similar variance in glycerol production has been observed for *L. thermotolerans* (Benito, 2018; Vicente et al., 2021). In pure *S. cerevisiae* fermentation, the final concentration of glycerol was 4.58 g/L. Conversely, simultaneous combinations of *L. thermotolerans* with lactic acid bacteria resulted in the lowest glycerol concentrations, ranging from 4.04 to 4.48 g/L.

In previous research, final glycerol concentrations were reported to increase by 0.59 g/L when comparing the traditional winemaking process—where alcoholic fermentation precedes malolactic fermentation (SC…OE)—to co-inoculation with *L. thermotolerans*, *O. oeni*, and *Saccharomyces* (LT*OE…SC) (Urbina et al., 2021). Contrasting results were observed in another study, with glycerol concentrations varying by *L. thermotolerans* strain: one strain raised glycerol levels by up to 2.6 g/L, while two other strains led to reductions ranging from 0.1 to 0.7 g/L (Snyder et al., 2021). The strain producing the least glycerol also generated the highest lactic acid concentration. In this study, a decrease of 0.57 g/L in glycerol was recorded (Table 2). This reduction was less pronounced, decreasing from 0.57 to 0.3 g/L, when *L. plantarum* (LT*LP…SC) was used in place of *O. oeni* (LT*OE…SC). Combining *L. thermotolerans* with *S. pombe* (LT…SP) resulted in a modest glycerol increase of 0.13 g/L compared to the traditional method (SC…OE). Other studies using similar biotechnological approaches (LT…SP) have reported either decreases in glycerol ranging from 0.64 to 2.64 g/L or increases from 0.27 to 0.71 g/L (Benito, 2020).

### 3.9 Color intensity

The most pronounced color intensities were observed in fermentations involving *S. pombe*. Earlier investigations into *S. pombe* attributed this observation to heightened Vitisin A production associated with pyruvic acid metabolism (Benito, 2019). Fermentations undergoing malolactic fermentation after alcoholic fermentation exhibited decreases in color intensity, ranging from 22 % in the case of the *S. cerevisiae* control to 24 % in combined fermentations involving *S. cerevisiae* and *L. thermotolerans*. Past studies have indicated that color intensity typically diminishes during malolactic fermentation (Benito, 2020). Fermentations involving *L. thermotolerans* showcased the lowest final color intensity (see Table 3). Previous research reported that while certain strains of *L. thermotolerans* can elevate color intensity by inducing substantial pH reductions that impact anthocyanin coloration, most strains also absorb notable concentrations of anthocyanins, contributing to a reduction in final color intensity (Benito, 2019; Benito, 2020).

**Table 3:** Final color parameters of fermentations: *S. cerevisiae* alone (SC); sequential fermentation with *S. cerevisiae* and *O. oeni* (SC….OE); sequential fermentation with *L. thermotolerans* and *S. cerevisiae* (LT…SC); sequential fermentation with *L. thermotolerans* and *S. cerevisiae*, followed by *O. oeni* (LT…SC….OE); sequential fermentation with *L. thermotolerans* and *S. pombe* (LT…SP); *S. pombe* alone (SP); sequential fermentation with *L. thermotolerans* and *O. oeni*, followed by *S. cerevisiae* (LT*OE…SC); sequential fermentation with *L. thermotolerans* and *L. plantarum*, followed by *S. cerevisiae* (LT*LP…SC); fermentation with *S. cerevisiae* followed by *O. oeni* 1 day after (SC*OE); fermentation with *S. cerevisiae* followed by *L. plantarum* 1 day after (SC*LP).

	SC	SC….OE	LT…SC	LT…SC….OE	LT*OE…SC	LT*LP…SC	SC*LP	SC*OE	SP	LT…SP
420 Absorbance	3.68 ± 0.08^a^	2.71 ± 0.02^de^	2.87 ± 0.12^cd^	2.23 ± 0.11^e^	2.71 ± 0.05^de^	2.73 ± 0.06^de^	3.58 ± 0.05^a^	3.37 ± 0.10^abc^	3.76 ± 0.05^a^	2.94 ± 0.30^bcd^
520 Absorbance	4.22 ± 0.11^ab^	3.23 ± 0.03^de^	3.70 ± 0.05^bcd^	2.91 ± 0.12^e^	3.35 ± 0.05^de^	3.48 ± 0.12^cde^	4.24 ± 0.08^ab^	3.76 ± 0.04^bcd^	4.49 ± 0.04^a^	3.57 ± 0.16^bcde^
620 Absorbance	0.74 ± 0.02^bc^	0.56 ± 0.02^ef^	0.62 ± 0.05^cde^	0.48 ± 0.02^f^	0.58 ± 0.01^def^	0.54 ± 0.03^ef^	0.72 ± 0.01^bc^	0.71 ± 0.01^bcd^	0.93 ± 0.02^a^	0.64 ± 0.08^cde^
Total polyphenol index	46.04 ± 4.44^abcd^	40.55 ± 0.49^e^	41.91 ± 1.28^de^	34.91 ± 0.43^f^	40.85 ± 0.38^e^	40.71 ± 0.33^e^	48.72 ± 0.64^a^	48.73 ± 0.65^a^	49.54 ± 0.18^a^	42.52 ± 1.08^cde^
Color Intensity (3)	8.64 ± 0.19^ab^	6.50 ± 0.05^ef^	7.20 ± 0.21^cde^	5.63 ± 0.25^f^	6.64 ± 0.09^def^	6.75 ± 0.18^def^	8.53 ± 0.12^ab^	7.84 ± 0.13^bcd^	9.18 ± 0.06^a^	7.14 ± 0.53^cde^
Color Intensity (2)	7.90 ± 0.18^ab^	5.94 ± 0.03^ef^	6.58 ± 0.16^cde^	5.15 ± 0.23^f^	6.06 ± 0.10^def^	6.21 ± 0.16^def^	7.81 ± 0.12^ab^	7.13 ± 0.13^abcd^	8.25 ± 0.06^a^	6.50 ± 0.45^cde^
Tonality	0.91 ± 0.02^ab^	0.87 ± 0.01^abcd^	0.82 ± 0.02^d^	0.83 ± 0.02^d^	0.84 ± 0.01^cd^	0.82 ± 0.01^d^	0.87 ± 0.01^bcd^	0.91 ± 0.01^ab^	0.86 ± 0.01^bcd^	0.86 ± 0.04^bcd^
^a^*	12.36 ± 13.17^a^	27.23 ± 5.11^a^	25.62 ± 3.29^a^	21.86 ± 13.83^a^	33.68 ± 1.10^a^	32.83 ± 4.40^a^	29.46 ± 3.31^a^	32.12 ± 4.57^a^	27.75 ± 3.08^a^	20.08 ± 13.12^a^
^b^*	4.70 ± 5.22^a^	12.36 ± 4.50^a^	10.00 ± 2.03^a^	10.48 ± 8.76^a^	18.99 ± 1.48^a^	17.86 ± 6.02^a^	13.81 ± 3.71^a^	17.34 ± 5.80^a^	11.89 ± 2.69^a^	9.40 ± 8.55^a^
C*	13.23 ± 14.16^a^	29.96 ± 6.48^a^	27.51 ± 3.80^a^	24.33 ± 16.17^a^	38.68 ± 1.69^a^	37.46 ± 6.79^a^	32.57 ± 4.56^a^	36.59 ± 6.70^a^	30.21 ± 3.87^a^	22.26 ± 15.48^a^
H*	20.01 ± 1.25^a^	23.83 ± 4.10^a^	21.18 ± 1.57^a^	23.23 ± 6.07^a^	29.39 ± 1.12^a^	27.99 ± 4.61^a^	24.78 ± 3.52^a^	27.74 ± 5.10^a^	22.99 ± 2.50^a^	22.59 ± 5.90^a^
L*	2.74 ± 3.04^a^	7.33 ± 2.67^a^	5.93 ± 1.22^a^	6.40 ± 5.36^a^	11.34 ± 0.85^a^	10.63 ± 3.60^a^	8.05 ± 2.17^a^	10.16 ± 3.38^a^	6.93 ± 1.56^a^	5.58 ± 5.09^a^
Total Anthocyanins (mg/L)	387.71 ± 10.11^b^	296.76 ± 2.76^f^	339.94 ± 4.59^c^	267.36 ± 11.02^g^	307.78 ± 4.59^de^	319.73 ± 11.03^d^	389.55 ± 7.35^b^	345.45 ± 3.68^c^	412.52 ± 3.68^a^	327.99 ± 14.70^cd^
Total Tannins (g/L)	1.91 ± 0.12^b^	1.73 ± 0.02^c^	1.76 ± 0.04^c^	1.48 ± 0.04^d^	1.73 ± 0.02^c^	1.72 ± 0.04^c^	2.05 ± 0.03^ab^	2.09 ± 0.03^a^	2.06 ± 0.01^a^	1.80 ± 0.06^bc^

Results are mean ± SD of three replicates. Different letters indicate statistical significance between groups.

Previous studies have shown a 21.58 % increase in final color intensity when comparing the traditional winemaking method—where alcoholic fermentation is followed by malolactic fermentation (SC…OE)—with a co-inoculation strategy involving *L. thermotolerans*, *O. oeni*, and *Saccharomyces* (LT*OE*…*SC*)* (Urbina et al., 2021). In the present study, color intensity increased by 2.11 % (Table 2), and this increase was further enhanced to 3.71 % (*v*/v) when *L. plantarum (*LT*LP…SC) replaced *O. oeni* (LT*OE…SC). Additionally, using *L. thermotolerans* in combination with *S. pombe* (LT…SP) resulted in a 9 % increase in color intensity over the traditional method (SC…OE). Other studies utilizing this approach have reported color intensity gains ranging from 7 % to 26 % (Benito, 2020).

### 3.10 Total anthocyanins and tannins

For total anthocyanins, the treatment with *S. pombe* showed the highest value, reaching 412.52 mg/L (Table 3). Compared to the pure fermentation with *S. cerevisiae*, which reached 387.71 mg/L, *S. pombe* exhibited an increase of 6.4 %. Among the treatments with intermediate values, the sequential fermentation of *L. thermotolerans* followed by *S. cerevisiae* yielded 339.94 mg/L, which represents a 12.3 % decrease compared to *S. cerevisiae*. On the other hand, the treatments with the lowest levels of anthocyanins were those with *S. cerevisiae* followed by *O. oeni*, which showed 296.76 mg/L, and the sequence of *L. thermotolerans*, *S. cerevisiae*, and then *O. oeni*, reaching 267.36 mg/L. This corresponds to reductions of 23.5 % and 31.0 % compared to *S. cerevisiae*, respectively. These variations suggest that treatments including *O. oeni*, particularly in long sequences, tend to significantly decrease the anthocyanin content.

Regarding total tannins, the richest treatments were those involving simultaneous fermentation of *S. cerevisiae* with *L. plantarum*, reaching 2.05 g/L, and *S. cerevisiae* with *O. oeni*, with 2.09 g/L, the latter being the highest value observed (Table 3). Compared to *S. cerevisiae* (1.91 g/L), simultaneous fermentation of *S. cerevisiae* with *O. oeni* had 9.4 % more tannins, while simultaneous fermentation of *S. cerevisiae* with *L. plantarum* exceeded *Saccharomyces cerevisiae* by 7.3 %. Intermediate treatments included *S. cerevisiae* with 1.91 g/L, followed by sequential treatments with *L. thermotolerans*, such as *L. thermotolerans* followed by *S. pombe* with 1.80 g/L, which is 5.8 % lower than *S. cerevisiae*. Finally, the treatment with the lowest tannin content was the sequential fermentation of *L. thermotolerans, S. cerevisiae*, and then *O. oeni*, reaching 1.48 g/L, showing a significant decrease of 22.5 % compared to *S. cerevisiae*.

In summary, treatments involving *S. pombe* and those where *S. cerevisiae* participates in simultaneous fermentation with *L. plantarum* or *O. oeni* significantly increase the phenolic compounds in wine, while sequential fermentations involving *L. thermotolerans* and *O. oeni* tend to reduce both anthocyanins and tannins. These variations in phenolic compounds may impact the sensory profile and quality of wine, contributing to differences in color, astringency, and antioxidant potential.

### 3.11 Higher alcohols

Fermentations involving *S. pombe* yielded the lowest final concentrations of the studied higher alcohols at 39.39 mg/L, while pure *S. cerevisiae* fermentation resulted in the highest concentration at 228 mg/L (Table 4), representing an 80 % decrease in higher alcohol production. Previous studies have reported reductions ranging from 22 % to 76 % in pure fermentations of *S. pombe* compared to *S. cerevisiae* (Benito, 2019; Benito, 2020). This phenomenon has previously been leveraged to prevent masking the varietal aroma profile of aromatic grape varieties. Hexanol was exclusively identified in trials involving *O. oeni*. Mixed simultaneous fermentations with *L. thermotolerans*, *O. oeni*, *L. plantarum*, and *S. cerevisiae* demonstrated the highest final concentrations of i-butanol, while pure *S. pombe* and combined fermentations involving *L. thermotolerans* and *S. pombe* exhibited the lowest concentrations. In terms of specific compounds, pure fermentation of *S. cerevisiae* registered the highest concentration of 3-methyl-butanol at 135 mg/L, whereas *S. pombe* exhibited the lowest at 21.71 mg/L. Sequential malolactic fermentations contributed to a reduction in the level of 3-methyl-butanol, with a similar effect observed for 2-methyl-butanol. Despite *S. pombe* yielding the lowest concentrations of higher alcohols, fermentations involving *S. pombe* and L. *thermotolerans* yielded higher concentrations of the specific higher alcohol, 2-phenyl-ethanol. Although pure *S. pombe* fermentation showed the lowest value in this compound (Table 4).

**Table 4:** Final volatile compound profiles of fermentations: *S. cerevisiae* alone (SC); sequential fermentation with *S. cerevisiae* and *O. oeni* (SC….OE); sequential fermentation with *L. thermotolerans* and *S. cerevisiae* (LT…SC); sequential fermentation with *L. thermotolerans* and *S. cerevisiae*, followed by *O. oeni* (LT…SC….OE); sequential fermentation with *L. thermotolerans* and *S. pombe* (LT…SP); *S. pombe* alone (SP); sequential fermentation with *L. thermotolerans* and *O. oeni*, followed by *S. cerevisiae* (LT*OE…SC); sequential fermentation with *L. thermotolerans* and *L. plantarum*, followed by *S. cerevisiae* (LT*LP…SC); fermentation with *S. cerevisiae* followed by *O. oeni* 1 day after (SC*OE); fermentation with *S. cerevisiae* followed by *L. plantarum* 1 day after (SC*LP).

Volatile Compound	SC	SC….OE	LT…SC	LT…SC….OE	LT*OE…SC	LT*LP…SC	SC*LP	SC*OE	SP	LT…SP
i-Butanol [mg/L]	31.83 ± 5.12^abc^	29.25 ± 2.11^abcd^	34.89 ± 3.67^ab^	34.41 ± 1.48^ab^	38.24 ± 2.48^a^	36.48 ± 1.92^ab^	29.05 ± 2.93^abcd^	27.57 ± 2.47^abcd^	6.52 ± 0.26^e^	16.54 ± 1.5^de^
3-Methyl-butanol [mg/L]	135.07 ± 15.23^a^	118.09 ± 5.97^a^	121.24 ± 5.64^a^	116.32 ± 1.13^a^	128.45 ± 9.87^a^	129.24 ± 4.2^a^	127.8 ± 6.14^a^	115.03 ± 6.22^a^	21.71 ± 2.29^c^	104.22 ± 11.91^a^
2-Methyl-butanol [mg/L]	40.65 ± 4.04^a^	36.52 ± 1.41^ab^	33.53 ± 1.91^ab^	33.72 ± 1.02^ab^	36.2 ± 2.46^ab^	38.09 ± 0.96^ab^	38.83 ± 3.32^ab^	37.43 ± 3.31^ab^	5.06 ± 0.43^d^	28.32 ± 2.38^bc^
2-Phenyl-ethanol [mg/L]	21.23 ± 3.36^a^	21.56 ± 1.16^a^	21.98 ± 0.7^a^	25.42 ± 2.51^a^	20.83 ± 0.49^a^	23.54 ± 1.37^a^	20.55 ± 0.7^a^	18.69 ± 1.32^a^	6.1 ± 0.13^b^	26.31 ± 5.05^a^
Hexanol [μg/L]	nd	306.57 ± 0.15^b^	nd	301.99 ± 1.43^b^	323.69 ± 9.97^a^	nd	nd	310.2 ± 6.3^ab^	nd	nd
Total Higher Alcohols [mg/L]	228.78	205.41	211.64	209.87	223.72	227.35	216.23	198.73	39.39	175.39

Propionic acid ethylester	132.95 ± 19.62^b^	117.25 ± 19.32^b^	574.64 ± 42.45^a^	522.45 ± 8.06^a^	495.54 ± 36.45^a^	529.71 ± 30.03^a^	117.65 ± 11.95^b^	102.9 ± 10.11^b^	102.5 ± 20.41^b^	553.37 ± 50.45^a^
[μg/L]										
Octanoic acid ethylester (Caprylic acid ethylester)	709.11 ± 217.56^ab^	358.97 ± 96.59^b^	nd	nd	nd	nd	650.31 ± 224.23^ab^	612.13 ± 97.64^b^	nd	nd
[μg/L]										
Decanoic acid ethylester (Capric acid ethylester) [μg/L]	390.03 ± 46.68^ab^	371.49 ± 36.59^b^	nd	nd	nd	nd	319.96 ± 37.41^b^	311.33 ± 12.36^b^	nd	nd
i-Butyric acid ethylester	20.02 ± 0.49^de^	19.66 ± 3.59^de^	39.61 ± 3.46^bc^	48.48 ± 1.4^ab^	36.63 ± 1.46^c^	35.12 ± 3.4^c^	24.76 ± 1.21^d^	12.9 ± 0.58^e^	23.67 ± 3.63^d^	39.55 ± 4.87^bc^
[μg/L]										
Butyric acid ethylester	251.85 ± 31.51^ab^	190.74 ± 20.34^abcd^	232.5 ± 42.39^ab^	209.07 ± 33.33^abc^	192.37 ± 3.07^abcd^	208.75 ± 15.85^abc^	244.74 ± 31.78^ab^	215.5 ± 19.78^abc^	86.48 ± 3.4^d^	95.57 ± 4.83^cd^
[μg/L]										
Lactic acid ethylester	28.9 ± 0.23^g^	52.55 ± 1.52^b^	43.63 ± 5.85^cde^	53.48 ± 3.35^b^	73.95 ± 3.75^a^	44.59 ± 1.52^bcd^	35.53 ± 1.32^efg^	52.89 ± 4.34^b^	28.27 ± 0.06^g^	34.05 ± 5.08^fg^
[mg/L]										
Total sters [mg/L]	30.4	53.05	44.47	54.25	73.72	45.36	36.88	54.14	28.48	34.73

Hexanoic acid (Caproic acid) [mg/L]	6.07 ± 0.23^ab^	6.16 ± 0.12^a^	5.58 ± 0.14^bc^	5.61 ± 0.14^bc^	5.43 ± 0.03^c^	5.46 ± 0.03^c^	6.19 ± 0.26^a^	6.05 ± 0.12^ab^	5.39 ± 0.01^c^	5.25 ± 0.02^c^
Hexanoic acid ethylester (Caproic acid ethylester) [μg/L]	428.73 ± 109.33^ab^	231.07 ± 58.24^bc^	193.78 ± 150.19^bc^	nd	18.03 ± 22.55^c^	nd	390.08 ± 117.64^abc^	305.86 ± 55.62^abc^	nd	nd
i-Valeric acid [μg/L]	1492.6 ± 93.78^a^	1566.57 ± 40.59^a^	nd	nd	nd	nd	1497.33 ± 21.22^a^	1452.43 ± 69.25^a^	nd	nd
Octanoic acid (Caprylic acid)	5.07 ± 0.6^a^	5.42 ± 0.19^a^	3.33 ± 0.20^b^	3.47 ± 0.23^b^	3.2 ± 0.05^b^	3.21 ± 0.02^b^	5.47 ± 0.54^a^	5.09 ± 0.19^a^	3.13 ± 0.02^b^	2.88 ± 0.02^b^
[mg/L]										
Decanoic acid (Capric acid) [μg/L]	1231.91 ± 155.8^a^	1413.03 ± 62.64^a^	nd	nd	nd	nd	1293.91 ± 142.05^a^	1170.45 ± 43.23^a^	nd	nd
Total Fatty Acids [mg/L]	14.29	14.79	9.10	9.08	8.64	8.67	14.84	14.06	8.52	8.13

Diacetyl (mg/L)	1.61 ± 0.11a	4.92 ± 0.26d	3.26 ± 0.20b	8.8 ± 1.75f	5.61 ± 0.33d	7.43 ± 1.35e	4.7 ± 0.62 cd	8.16 ± 1.11ef	0.53 ± 0.11a	3.36 ± 0.21bc

Results are mean ± SD of three replicates. Different letters indicate statistical significance between groups. nd: below the detection limit.

A previous study reported no statistically significant differences in the primary higher alcohols, 2-methyl-butanol and 3-methyl-butanol, when comparing the traditional winemaking method—where alcoholic fermentation is completed first, followed by malolactic fermentation (SC…OE)—with co-inoculation of *L. thermotolerans*, *O. oeni*, and *Saccharomyces* (LT*OE…SC) (Urbina et al., 2021). The same outcome was observed in this study (Table 4). Here, reductions in 2-methyl-butanol and 3-methyl-butanol of 22.5 % and 12 %, respectively, were recorded for the combined use of *L. thermotolerans* and *S. pombe* (LT…SP) compared to the traditional method (SC…OE). Previous studies using this biotechnology have reported reductions in 2-methyl-butanol and 3-methyl-butanol ranging from 21 % to 35 % and from 7 % to 24 %, respectively (Benito, 2020).

### 3.12 Esters

Fermentations involving *L. thermotolerans* or *S. pombe* in various combinations did not yield detectable concentrations of octanoic acid ethyl ester or decanoic acid ethyl ester (Table 4). Sole fermentation by *S. cerevisiae* resulted in the highest concentration of octanoic acid ethyl ester, while combinations with lactic acid bacteria led to lower concentrations, particularly in the case of *O. oeni* combinations. Fermentations involving *L. thermotolerans* produced significantly higher concentrations of propionic acid ethyl ester, approximately five times higher than observed in fermentations of *S. cerevisiae*, *S. cerevisiae* combined with lactic acid bacteria, and *S. pombe*. A similar trend was observed for i-butyric acid ethyl ester, albeit with an approximate one-time increase. Pure fermentation of *S. cerevisiae* and its combination with *L. plantarum* exhibited the highest production of butyric acid ethyl ester, while *S. pombe* displayed the lowest yield. Combinations involving *S. cerevisiae* with *O. oeni* and *L. thermotolerans* contributed to a reduction in butyric acid ethyl ester. Fermentations involving microorganisms capable of producing lactic acid, such as *L. thermotolerans*, *O. oeni*, or *L. plantarum*, showed higher final concentrations of lactic acid ethyl ester compared to yeasts lacking this metabolic pathway.

In previous research, final lactic acid ethyester concentrations were reported to increase by 42 % when comparing the traditional winemaking process—where alcoholic fermentation precedes malolactic fermentation (SC…OE)—to co-inoculation with *L. thermotolerans*, *O. oeni*, and *Saccharomyces* (LT*OE…SC) (Urbina et al., 2021). This study observed and increased in 29 %.

### 3.13 Fatty acids

In the absence of non-*Saccharomyces* influence, pure fermentations of *S. cerevisiae* exhibited the highest concentrations of hexanoic acid, a trend also observed for octanoic acid. Fermentations involving *L. thermotolerans* or *S. pombe* did not yield detectable levels of i-valeric acid, mirroring the outcome for decanoic acid.

### 3.14 Diacetyl

Pure fermentations of *S. pombe* and *S. cerevisiae* showed the lowest final concentrations in diacetyl. The combination of these species with *L. thermotolerans* slightly increased the final concentration of diacetyl. The highest final concentrations were related to the combinations involving lactic acid bacteria (Table 4) that showed final values that varied from 4 to 8 times the concentration of the pure controls of *S. cerevisiae* and *S. pombe*.

The traditional malolactic fermentation (SC…OE) produced an increase in diacetyl of 3.31 mg/L compared to the pure control of *S. cerevisiae* that only performed alcoholic fermentation (SC) (Table 4). The co-inoculation between *L. thermotolerans*, *O. oeni*, and *Saccharomyces* (LT*OE…SC) showed no statistical differences with the traditional methodology while when the bacterium *L. plantarum* (LT*LP…SC) was used the increase was significant in 2.51 mg/L. The combined use of *L. thermotolerans* and *S. pombe* (LT…SP) produced les Diacetyl than the traditional malolactic fermentation (SC…OE) in 1.56 mg/L.

### 3.15 Executive summary

The results from this study underscore the potential of alternative microbial combinations in addressing the challenges posed by traditional alcoholic fermentation followed by malolactic fermentation, particularly in warm viticultural regions affected by climate change. Elevated temperatures in such regions often lead to grape musts with lower acidity, higher sugar concentrations, and nutrient deficiencies, which result in sluggish or stalled fermentations and an increased risk of spoilage by undesirable bacteria. The study demonstrates that the use of combined fermentations can alleviate these issues, offering benefits such as ethanol reduction, malic acid consumption, lactic acid production, and overall improvements in wine quality and characteristics.

The combined use of *L. thermotolerans* with *O. oeni* followed by *S. cerevisiae* (LT*OE…SC) has proven to be an effective strategy for managing these challenges. This approach significantly lowers pH and increases total acidity, making it particularly suitable for enhancing wine freshness in regions where acidity is often compromised. The role of *O. oeni* in ensuring the complete degradation of malic acid early in fermentation stabilises the wine microbiologically and prevents the risk of spontaneous malolactic fermentation, which is more likely to fail under high-temperature conditions. Although a slight increase in acetic acid production is observed, it remains below levels considered problematic, making LT*OE…SC an optimal solution for winemakers seeking to improve acidity and microbial stability in the face of climate-induced fermentation difficulties.

Alternatively, sequential fermentation involving *L. thermotolerans* and *S. pombe* (LT…SP) offers a promising solution. This strategy reduces ethanol content, which is advantageous as climate change leads to higher sugar concentrations in grapes, consequently increasing alcohol levels in wine. Furthermore, LT…SP demonstrates near-complete malic acid consumption, contributing to lower final acetic acid concentrations. This method also results in higher color intensity, which can be particularly desirable in certain wine styles. While the reduction in pH and increase in total acidity is somewhat less pronounced compared to LT*OE…SC, LT…SP offers a balanced approach for wines aiming for lower alcohol content and enhanced color.

Comparing the final volatile compound profiles of LT*OE…SC and LT…SP reveals significant differences in aromatic complexity and intensity. The LT*OE…SC combination yields higher concentrations of esters and higher alcohols, contributing to a more complex and intense aroma. Lactic acid ethyl ester (73.95 mg/L) and propionic acid ethyl ester (495.54 μg/L) are elevated, enhancing the fruity and complex aromatic profile, while the presence of hexanol (323.69 μg/L) introduces fresh, green notes. Higher levels of 3-methyl-butanol and isobutanol also contribute to a more robust aromatic intensity, though they may risk producing heavier aromas. In contrast, LT…SP produces fewer esters overall (34.73 mg/L) and a lower total of higher alcohols, resulting in a lighter, more refined aromatic profile. However, it excels in propionic acid ethyl ester production (553.37 μg/L), further enhancing fruity characteristics. The absence of hexanol avoids herbal notes, allowing fruit aromas to emerge more clearly. Therefore, LT*OE…SC is likely to yield a more complex and intense aroma, while LT…SP offers a cleaner, fruit-forward profile with a lighter aromatic footprint.

In terms of phenolic content, the LT*OE…SC treatment (simultaneous fermentation of *L. thermotolerans* and *O. oeni,* followed by *S. cerevisiae*) yielded a total anthocyanin concentration of 307.78 mg/L, while LT…SP (sequential fermentation with *S. pombe*) reached 327.99 mg/L, representing a significant 6.2 % increase. This indicates that the sequential fermentation with *S. pombe* better preserves anthocyanins, contributing to stronger color retention. Similarly, LT*OE…SC reached 1.73 g/L of total tannins, while LT…SP displayed a higher tannin content of 1.80 g/L, a 4.0 % difference. This suggests that the sequential combination with *S. pombe* contributes to greater tannin retention, resulting in wines with more intense color and astringency.

Overall, the LT…SP treatment demonstrated a significantly higher phenolic profile, both in anthocyanins and tannins, when compared to LT*OE…SC. These higher levels in LT…SP suggest that this method could produce wines with more intense color and greater astringency, traits that are highly valued in certain wine styles. In contrast, LT*OE…SC, which incorporates *O. oeni* in a simultaneous fermentation with *L. thermotolerans* and *S. cerevisiae*, exhibited a significant reduction in both anthocyanins and tannins, potentially resulting in a wine with a softer, less astringent profile.

In conclusion, the most appropriate microbial strategies to address the challenges of traditional malolactic fermentation under climate change conditions include the combined use of *L. thermotolerans* with *O. oeni* followed by *S. cerevisiae* (LT*OE…SC*)* and sequential fermentation involving *L. thermotolerans* and *S. pombe (*LT…SP). LT*OE…SC is optimal for optimizing acidity, microbial stability, and overall freshness, particularly in regions where low acidity is a concern. It may also enhance aroma complexity through higher ester and alcohol production. LT…SP is well-suited for controlling ethanol levels, reducing volatile acidity, and producing wines with enhanced color and a lighter, fruit-forward aromatic profile. Moreover, LT…SP helps preserve a higher amount of anthocyanins and tannins, contributing to more intense color and greater astringency. These biotechnological strategies offer winemakers valuable tools to adapt to the ongoing challenges posed by climate change and ensure the stability and quality of wines produced under increasingly difficult conditions.

## 4 Conclusion

This study highlights the potential of alternative microbial combinations to address the challenges faced by traditional malolactic fermentation, particularly in warm viticultural regions affected by climate change. The findings confirm that both LT*OE…SC (sequential fermentation with *L. thermotolerans*, *O. oeni,* and *S. cerevisiae*) and LT…SP (sequential fermentation with *L. thermotolerans* and *S. pombe*) offer viable solutions for optimizing wine fermentation under these conditions. LT*OE…SC proves to be an excellent option for regions where acidity is compromised due to high temperatures. It significantly reduces pH and increases total acidity, enhancing the freshness and microbial stability of the wine. Furthermore, its complex volatile compound profile, characterised by higher ester and alcohol production, may provide greater aromatic complexity and intensity, with fruity and fresh green notes. This combination could be ideal for winemakers aiming to produce wines with a strong aromatic profile and enhanced acidity in warmer climates. On the other hand, LT…SP excels in scenarios where ethanol reduction is a priority, as it effectively lowers alcohol content while maintaining malic acid stabilization. Although it produces fewer esters and higher alcohols compared to LT*OE…SC, its higher concentration of propionic acid ethyl ester could contribute to a clean, fruit-forward aroma. The absence of herbal notes like hexanol may enhance the focus on the fruity character, making this combination suitable for lighter, more delicate wine styles.

In conclusion, both combinations present viable strategies for overcoming the fermentation challenges induced by climate change. LT*OE…SC is particularly suited for enhancing acidity and creating wines with a robust aromatic profile, while LT…SP offers a balanced solution for reducing ethanol and emphasising fruit-forward aromas. Winemakers can choose the most appropriate combination depending on their goals, whether focusing on acidity, aroma complexity, or alcohol reduction, ensuring high-quality wine production in increasingly warm climates.

## Author contributions

S.Be., J.V., D.M., D.R., and L.W. developed the experimental design; J.V., L.W., W. T., D.M., A.S., E.N., and S.Be. performed the vinifications; J.V., L.W., and S.Be. performed the formal data analysis and supervised the project; J.V., L.W., and S.Be. wrote the article; D.R., S.Br. and S.F. performed gas chromatographic analysis. J.V., L.W., F.C., W.T., and S.Be performed enzymatic and FTIR analysis. All authors discussed the results and contributed to the final manuscript.

## CRediT authorship contribution statement

**Javier Vicente:** Writing – review & editing, Writing – original draft, Project administration, Methodology, Investigation, Formal analysis. **Li Wang:** Writing – review & editing, Writing – original draft, Methodology, Investigation, Formal analysis, Data curation, Conceptualization. **Silvia Brezina:** Methodology, Formal analysis. **Stefanie Fritsch:** Methodology, Formal analysis. **Eva Navascués:** Supervision, Methodology, Funding acquisition, Conceptualization. **Antonio Santos:** Writing – review & editing, Supervision, Resources, Methodology, Conceptualization. **Fernando Calderón:** Writing – review & editing, Supervision, Formal analysis. **Wendu Tesfaye:** Writing – review & editing, Supervision, Resources, Formal analysis. **Domingo Marquina:** Writing – review & editing, Supervision, Resources, Project administration, Methodology, Funding acquisition, Conceptualization. **Doris Rauhut:** Writing – review & editing, Supervision, Resources, Methodology, Investigation, Funding acquisition, Formal analysis. **Santiago Benito:** Writing – review & editing, Writing – original draft, Visualization, Validation, Supervision, Software, Resources, Project administration, Methodology, Investigation, Funding acquisition, Formal analysis, Data curation, Conceptualization.

## Declaration of competing interest

The authors declare that they have no known competing financial interests or personal relationships that could have appeared to influence the work reported in this paper.

## Data Availability

Data will be made available on request.

## References

[bb0005] Bartowsky E.J., Costello P.J., Chambers P.J. (2015). Emerging trends in the application of malolactic fermentation. Australian Journal of Grape and Wine Research.

[bb0010] Benito S. (2018). The impacts of *Lachancea thermotolerans* yeast strains on winemaking. Applied Microbiology and Biotechnology.

[bb0015] Benito S. (2019). The impacts of *Schizosaccharomyces* on winemaking. Applied Microbiology and Biotechnology.

[bb0020] Benito S. (2020). Combined use of *Lachancea thermotolerans* and *Schizosaccharomyces pombe* in winemaking: A review. Microorganisms.

[bb0025] Blanco P., Rabuñal E., Neira N., Castrillo D. (2020). Dynamic of *Lachancea thermotolerans* population in monoculture and mixed fermentations: Impact on wine characteristics. Beverages.

[bb0030] Borren E., Tian B. (2020). The important contribution of non-*Saccharomyces* yeasts to the aroma complexity of wine: A review. Foods.

[bb0035] Capozzi V., Tufariello M., De Simone N., Fragasso M., Grieco F. (2021). Biodiversity of oenological lactic acid Bacteria: Species- and strain-dependent plus/minus effects on wine quality and safety. Fermentation.

[bb0040] Chen K., Escott C., Loira I., del Fresno J.M., Morata A., Tesfaye W., Calderon F., Suárez-Lepe J.A., Han S., Benito S. (2018). Use of non-*Saccharomyces* yeasts and oenological tannin in red winemaking: Influence on colour, aroma and sensorial properties of young wines. Food Microbiology.

[bb0045] Ciani M., Beco L., Comitini F. (2006). Fermentation behavior and metabolic interactions of multistarter wine yeast fermentations. International Journal of Food Microbiology.

[bb0050] Comitini F., Gobbi M., Domizio P., Romani C., Lencioni L., Mannazzu I., Ciani M. (2011). Selected non-*Saccharomyces* wine yeasts in controlled multistarter fermentations with *Saccharomyces cerevisiae*. Food Microbiology.

[bb0055] Domizio P., Liu Y., Bisson L.F., Barile D. (2014). Use of non-*Saccharomyces* wine yeasts as novel sources of mannoproteins in wine. Food Microbiology.

[bb0060] Du Plessis H., Du Toit M., Nieuwoudt H., Van der Rijst M., Hoff J., Jolly N. (2019). Modulation of wine flavor using *Hanseniaspora uvarum* in combination with different *Saccharomyces cerevisiae*, lactic acid bacteria strains, and malolactic fermentation strategies. Fermentation.

[bb0065] Du Toit M., Engelbrecht L., Lerm E., Krieger-Weber S. (2011). *Lactobacillus*: The next generation of malolactic fermentation starter cultures—An overview. Food and Bioprocess Technology.

[bb0070] Escribano R., González-Arenzana L., Portu J., Garijo P., López-Alfaro I., López R., Santamaría P., Gutiérrez A.R. (2018). Wine aromatic compound production and fermentative behavior within different non-*Saccharomyces* species and clones. Journal of Applied Microbiology.

[bb0075] Fairbairn S., Engelbrecht L., Setati M.E., du Toit M., Bauer F.F., Divol B., Rossouw D. (2021). Combinatorial analysis of population dynamics, metabolite levels, and malolactic fermentation in *Saccharomyces cerevisiae/Lachancea thermotolerans* mixed fermentations. Food Microbiology.

[bb0080] Gardoni E., Benito S., Scansani S., Brezina S., Fritsch S., Rauhut D. (2021). Biological deacidification strategies for white wines. South African Journal of Enology and Viticulture.

[bb0085] Gobbi M., Comitini F., Domizio P., Romani C., Lencioni L., Mannazzu I., Ciani M. (2013). *Lachancea thermotolerans* and *Saccharomyces cerevisiae* in simultaneous and sequential co-fermentation: A strategy to enhance acidity and improve the overall quality of wine. Food Microbiology.

[bb0090] Hranilovic A., Albertin W., Capone D.L., Gallo A., Grbin P.R., Danner L., Jiranek V. (2021). Impact of *Lachancea thermotolerans* on chemical composition and sensory profiles of Merlot wines. Food Chemistry.

[bb0095] Hranilovic A., Albertin W., Capone D.L., Gallo A., Grbin P.R., Danner L., Jiranek V. (2022). Impact of *Lachancea thermotolerans* on chemical composition and sensory profiles of viognier wines. Journal of Fungus.

[bb0100] Hranilovic A., Li S., Boss P.K., Bindon K., Ristic R., Grbin P.R., Van der Westhuizen T., Jiranek V. (2018). Chemical and sensory profiling of Shiraz wines co-fermented with commercial non-*Saccharomyces* inocula. Australian Journal of Grape and Wine Research.

[bb0105] Jolly N.P., Varela C., Pretorius I.S. (2014). Not your ordinary yeast: Non-*Saccharomyces* yeasts in wine production uncovered. FEMS Yeast Research.

[bb0110] Jung R., Kumar K., Patz C., Rauhut D., Tarasov A., Schüßler C. (2021). Influence of transport temperature profiles on wine quality. Food Packaging and Shelf Life.

[bb0115] Kapsopoulou K., Mourtzini A., Anthoulas M., Nerantzis E. (2007). Biological acidification during grape must fermentation using mixed cultures of *Kluyveromyces thermotolerans* and *Saccharomyces cerevisiae*. World Journal of Microbiology and Biotechnology.

[bb0120] Knoll C., Fritsch S., Schnell S., Grossmann M., Krieger-Weber S., Du Toit M., Rauhut D. (2012). Impact of different malolactic fermentation inoculation scenarios on Riesling wine aroma. World Journal of Microbiology and Biotechnology.

[bb0125] Krieger-Weber S., Heras J.M., Suarez C. (2020). *Lactobacillus plantarum*, a new biological tool to control malolactic fermentation: A review and an outlook. Beverages.

[bb0130] Mendes Ferreira A., Mendes-Faia A. (2020). The role of yeasts and lactic acid Bacteria on the metabolism of organic acids during winemaking. Foods.

[bb0135] Pardo I., Ferrer S. (2018). Red wine technology.

[bb0140] Petruzzi L., Capozzi V., Berbegal C., Corbo M.R., Bevilacqua A., Spano G., Sinigaglia M. (2017). Microbial resources and enological significance: Opportunities and benefits. Frontiers in Microbiology.

[bb0145] Pinto L., Baruzzi F., Cocolin L., Malfeito-Ferreira M. (2020). Emerging technologies to control Brettanomyces spp. in wine: Recent advances and future trends. Trends in Food Science & Technology.

[bb0150] Porter T.J., Divol B., Setati M.E. (2019). Investigating the biochemical and fermentation attributes of *Lachancea* species and strains: Deciphering the potential contribution to wine chemical composition. International Journal of Food Microbiology.

[bb0155] Porter T.J., Divol B., Setati M.E. (2019). *Lachancea* yeast species: Origin, biochemical characteristics, and oenological significance. International Food Research.

[bb0160] Ribereau-Gayon P., Glories Y., Maujean A., Dubourdieu D., Ribereau-Gayon P., Glories Y., Maujean A., Dubourdieu D. (2006). Handbook of enology.

[bb0165] Snyder E.C., Jiranek V., Hranilovic A. (2021). Impact of *Lachancea thermotolerans* strain and lactic acid concentration on *Oenococcus oeni* and malolactic fermentation in wine. OENO one.

[bb0170] Sumby K.M., Grbin P.R., Jiranek V. (2014). Implications of new research and technologies for malolactic fermentation in wine. Applied Microbiology and Biotechnology.

[bb0175] Urbina Á., Calderón F., Benito S. (2021). The combined use of *Lachancea thermotolerans* and *Lactiplantibacillus plantarum* (formerly *Lactobacillus plantarum*) in wine technology. Foods.

[bb0180] Vicente J., Baran Y., Navascués E., Santos A., Calderón F., Marquina D., Rauhut D., Benito S. (2022). Biological management of acidity in wine industry: A review. International Journal of Food Microbiology.

[bb0185] Vicente J., Kelanne N., Navascués E., Calderón F., Santos A., Marquina D., Yang B., Benito S. (2023). Combined use of *Schizosaccharomyces pombe* and a *Lachancea thermotolerans* strain with a high malic acid consumption ability for wine production. Fermentation.

[bb0190] Vicente J., Navascués E., Calderón F., Santos A., Marquina D., Benito S. (2021). An integrative view of the role of *Lachancea thermotolerans* in wine technology. Foods.

[bb0195] Vicente J., Vladic L., Navascués E., Brezina S., Santos A., Calderón F., Tesfaye W., Marquina D., Rauhut D., Benito S. (2024). A comparative study of *Lachancea thermotolerans* fermentative performance under standardized wine production conditions. Food Chemistry: X.

[bb0200] Vilela A. (2018). *Lachancea thermotolerans*, the non-*Saccharomyces* yeast that reduces the volatile acidity of wines. Fermentation.

[bb0205] Vilela A. (2019). Use of nonconventional yeasts for modulating wine acidity. Fermentation.

[bb0210] Virdis C., Sumby K., Bartowsky E., Jiranek V. (2021). Lactic acid Bacteria in wine: Technological advances and evaluation of their functional role. Frontiers in Microbiology.

[bb0215] Wang Y., Sheng W., Li M., Mi L., Jiang Y. (2019). Effect of sequential fermentation with *Lachancea thermotolerans* and *Schizosaccharomyces pombe* on the quality of merlot dry red wine. Food Science.

